# Early hemodynamic changes after the use of push-dose noradrenaline in patients presenting to the emergency department with septic shock: a descriptive study

**DOI:** 10.1186/s12873-026-01773-8

**Published:** 2026-09-19

**Authors:** Atsushi Yamamoto, Tomoyuki Akita, Aya Sugiyama, Sayaka Nakahara, Yuhei Urakami, Soichiro Mae, Tetsunori Ikegami, Junko Tanaka

**Affiliations:** 1 Emergency and Critical Care Center, Kurashiki Central Hospital, Okayama, Japan; 2 Department of Epidemiology, Disease Control and Prevention, Graduate School of Biomedical and Health Sciences, Hiroshima University, Hiroshima, Japan; 3 Project Research Center for Epidemiology and Prevention of Viral Hepatitis and Hepatocellular Carcinoma, Hiroshima University, Hiroshima, Japan

**Keywords:** Emergency service, Hospital, Hypotension, Noradrenaline, Shock, Vasoconstrictor agents

## Abstract

**Background:**

Although continuous noradrenaline infusion (CNI) is standard vasopressor therapy during the initial resuscitation of patients with septic shock, hemodynamic stabilization may not be immediate. Push-dose noradrenaline (PDNA) has been proposed as a temporary measure while CNI is being established. However, its clinical use, dosing, early hemodynamic response, and short-term safety in the emergency department (ED) are not well described.

**Methods:**

To address this gap, we conducted a single-center retrospective descriptive study at a tertiary care hospital in Japan in 2019. Adults with Sepsis-3 septic shock who received PDNA in the ED and were admitted to the intensive care unit were included. We assessed the context and dosing of PDNA administration, CNI use, mean arterial pressure (MAP) within 15 min after PDNA administration, and adverse events.

**Results:**

Of 91 patients with septic shock, 50 (55%) received PDNA. PDNA was administered at CNI initiation in 25 patients, during CNI dose escalation in 6, and as rescue administration before CNI initiation in 19. The median (interquartile range [IQR]) initial PDNA dose was 20 (20–30) µg. During the 15-minute observation window, 32 (64%) patients received one bolus, 14 (28%) received two, 3 (6%) received three, and 1 (2%) received four. The directly observed median (IQR) MAP was 52 (44–61) mmHg at initial PDNA administration (*n* = 30) and 62 (50–68) mmHg 1 min later (*n* = 25). One of 36 patients evaluable for prespecified physiological adverse events experienced an adverse event; however, no serious adverse events were documented.

**Conclusions:**

In this single-center retrospective descriptive study, PDNA was most commonly administered at CNI initiation, and administration was followed by an early increase in median MAP. However, the noncomparative design and concurrent resuscitative interventions preclude attribution of this change solely to PDNA administration. No serious adverse events were documented; however, safety assessment was limited owing to missing data and retrospective ascertainment. These findings do not establish the effectiveness or safety of PDNA. Prospective comparative studies with standardized monitoring are required.

**Supplementary Information:**

The online version contains supplementary material available at https://doi.org/10.1186/s12873-026-01773-8.

## Background

Septic shock is common among patients presenting to the emergency department (ED) and requires prompt recognition and treatment. In patients with septic shock, delayed vasopressor initiation is associated with worse outcomes [1], and even transient hypotension can contribute to increased mortality [2], highlighting the importance of early hemodynamic stabilization.

Although continuous noradrenaline infusion (CNI) is the standard vasopressor therapy for septic shock, adequate blood pressure control may not be achieved immediately [3]. In real-world ED practice, CNI may not be available at the time of a hypotensive emergency [4]. Moreover, adequate blood pressure control may not be achieved immediately after CNI initiation because vasopressor titration takes time [5]. Recent intensive care unit (ICU) data suggest that the early mean arterial pressure (MAP) response to CNI is dose dependent. In modeled analyses of starting doses of 0.025, 0.050, and 0.100 µg/kg/min, MAP generally approached a plateau approximately 45–90 min after initiation, with higher starting doses leading to earlier stabilization [5]. Thus, even after CNI is initiated, achieving adequate hemodynamic support may take time.

The administration of push-dose pressors has been proposed as a temporary measure for blood pressure support during this vulnerable period; however, evidence for their use in non-operating room settings is derived predominantly from observational studies, and the optimal agent, dose, and monitoring strategy in the ED remain unclear [6]. Push-dose noradrenaline (PDNA) may be a practical option because of its hemodynamic profile and compatibility with ongoing noradrenaline infusion [7]. However, information on how PDNA is used during septic shock resuscitation in the ED setting remains limited.

Although noradrenaline boluses have been widely studied in perioperative settings [7], those findings may not be applicable to patients with septic shock in the ED setting. The clinical characteristics, monitoring conditions, and operational constraints of patients with septic shock in nonoperative settings differ substantially from those of patients in operative settings. In the ED setting, the timing and dosing patterns of PDNA use, early MAP trajectories, and adverse events remain poorly characterized [6, 7]. To provide information on real-world practice and address this knowledge gap, we conducted a study to characterize the clinical context of PDNA use in patients presenting to the ED with septic shock and describe early MAP trajectories after administration and adverse events.

## Methods

### Study design and setting

This retrospective descriptive study was conducted at a tertiary care teaching hospital in western Japan. The ED treats approximately 60,000 patients annually. We analyzed data from a full one-year cohort of patients treated in the ED between January 1 and December 31, 2019. This period was chosen to enable analysis of PDNA administration data and available early hemodynamic measurements in a stable pre-COVID-19 clinical environment. This study was reported in accordance with the Strengthening the Reporting of Observational Studies in Epidemiology (STROBE) statement [8]. The ethics committee of Kurashiki Central Hospital approved the study (approval no. 3699) and waived the requirement for informed consent owing to the retrospective design and use of pseudonymized data.

### Patients

Patients were eligible if they were adults (aged ≥ 18 years) who presented to the ED with septic shock, received both PDNA and continuous vasopressor infusion in the ED, and were admitted to the ICU from the ED. For this study, septic shock was operationally defined as a documented clinical diagnosis of septic shock in the electronic medical record (EMR). In addition, patients were required to meet the following criteria after initial fluid resuscitation: vasopressor therapy was required to maintain a MAP ≥ 65 mmHg, and serum lactate levels were ≥ 2 mmol/L. These criteria were based on the vasopressor and lactate components of the Sepsis-3 definition [3]. The adequacy of initial fluid resuscitation was not assessed retrospectively using a standardized definition. Fluid administration and the timing of vasopressor initiation reflected routine clinical practice and were determined by the treating physicians based on their overall clinical assessment; however, the specific basis for these decisions was not consistently documented and could not be systematically ascertained for all patients. PDNA was defined as an intravenous bolus administration of any dose of noradrenaline. Patients who received PDNA were identified from electronic medication administration records, and eligibility was confirmed through manual review of their EMRs. Their clinical characteristics, patterns of PDNA use, and early MAP trajectories after administration were described.

Patients were excluded if (i) they were admitted to a cardiovascular or stroke-specific ICU that was not captured in the ICU database, (ii) continuous vasopressor infusion was initiated before arrival (e.g., started at another facility), (iii) they received other push-dose vasopressors during the observation period, or (iv) essential clinical data were missing from their medical records. All eligible patients were included. No a priori sample size calculation was performed.

### Noradrenaline formulation, preparation, and administration

According to recent recommendations for standardizing noradrenaline dose reporting, all noradrenaline doses were reported as noradrenaline base equivalents [9]. Noradrenaline was prepared immediately before administration in the ED by a nurse, physician, or pharmacist by adding 1 mg of noradrenaline to a 100-mL bag of normal saline, yielding a solution of approximately 10 µg/mL. This solution was used for both PDNA and CNI according to institutional practice. All PDNA doses were administered through peripheral venous catheters via a running intravenous line. No standardized flushing protocol was documented, and whether flushing was performed after each administration could not be reliably ascertained retrospectively. As part of routine institutional practice, the drug, dilution, concentration, and intended dose were independently double-checked by another healthcare professional. Peripheral intravenous catheter patency and the insertion site were routinely assessed as part of clinical care for signs of infiltration or extravasation; however, these assessments were not necessarily documented for every administration. Potential preparation or administration errors were assessed by reviewing EMRs and institutional safety reports. In our ED, the only push-dose vasopressors available were noradrenaline and adrenaline.

PDNA was administered at the discretion of the treating physician during early resuscitation in the ED. The clinical context of PDNA use was described and categorized into three predefined groups according to its temporal relationship with CNI management: administration at CNI initiation, administration during CNI dose escalation, and rescue administration before CNI initiation. These predefined categories were designed to describe common clinical situations in which early vasopressor management is used rather than to describe the specific treatment indication. Endotracheal intubation was considered temporally associated with PDNA if it was performed within the interval from 10 min before to 5 min after the initial dose. The number, dose, and timing of PDNA and CNI administrations, as well as concurrent interventions, were also recorded.

### Data sources and measurements

Hemodynamic and treatment-related data were collected to describe early clinical trajectories during vasopressor management. Time-series data, including vital signs and medication administration times, were extracted from the electronic patient management system in the ED (ACSYS-Ki; Philips Japan, Tokyo, Japan). Medication timing variables were based on time-stamped administration records rather than order-entry times. Additional clinical data, including diagnosis, shock classification, Acute Physiology and Chronic Health Evaluation (APACHE) II score [10], and serum lactate levels during the ICU stay, were obtained from the EMR and ICU database.

Blood pressure measurements were obtained as part of routine clinical care using either automated noninvasive cuff measurements or arterial lines when available. When both modalities were present, arterial-line measurements were used preferentially. If MAP was not directly available in the dataset, it was calculated from the diastolic blood pressure (DBP) and systolic blood pressure (SBP) as follows:

*DBP+(SBP-DBP)/3 *

Baseline hemodynamic severity and concurrent interventions were identified from time-stamped records [11]. Shock classification was determined based on ED documentation and the final ICU diagnosis, supported by routine clinical and ultrasonographic evaluation, as appropriate. Missing data were not imputed in this study.

### Outcomes

Serial MAP values during the 15 min after the initial PDNA administration were recorded and summarized at prespecified 1-minute intervals. At each time point, the median MAP and interquartile range (IQR) were calculated using all available directly observed measurements for that specific minute; therefore, the number of patients contributing measurements and the sample size varied between time points. If no blood pressure measurement was available at a given time point, data for that time point were treated as missing. Missing MAP values were not imputed, interpolated, or replaced with measurements from the nearest time point. Because measurements were obtained during routine clinical care, not all patients had data available at every time point; therefore, the number of patients contributing data was reported for each time point.

Prespecified adverse events were defined according to definitions used in previous studies [12]. Prespecified physiological adverse events were based on recorded blood pressure and heart rate (HR) measurements within 5 min after the initial PDNA administration. Other prespecified adverse events and medication errors were ascertained by chart review during hospitalization, unless otherwise specified. The following prespecified adverse events were recorded after the initial PDNA administration: (1) severe hypertension or marked SBP increase within 5 min: SBP > 200 mmHg or an increase in SBP of > 100% from baseline; (2) hypertension requiring treatment: initiation of antihypertensive therapy due to elevated blood pressure, as documented in the EMR; (3) HR change: a relative change in HR of > 30% within 5 min (either increase or decrease); (4) ventricular tachycardia; (5) vasospasm; (6) acute myocardial infarction within 24 h; (7) new troponin elevation within 24 h; and (8) skin necrosis. Baseline HR was defined as the last recorded value before the initial PDNA administration, and an HR event was considered present if any measurement within 5 min after PDNA administration met the prespecified threshold. Vasospasm was defined as either a clinically diagnosed condition documented in the EMR or vasospasm confirmed by catheter examination and documented in the EMR. Acute myocardial infarction within 24 h was defined as a catheter-confirmed diagnosis documented in the EMR. For time-limited events (acute myocardial infarction and new troponin elevation), the 24-hour window was defined from the time of initial PDNA administration in the ED. Medication errors were defined according to a previous study [13] as either a documented medication error in the EMR or a medical safety report indicating a medication error.

### Data analysis

We conducted a descriptive analysis of serial MAP values after PDNA administration. Continuous variables are reported as medians with IQRs, and categorical variables are reported as counts and percentages. For serial measurements, the number of patients contributing data was reported at each time point. Exploratory paired analyses were performed using the Wilcoxon signed-rank test to compare MAP at PDNA initiation with MAP at 1, 5, 10, and 15 min after administration. For each comparison, all patients with directly observed MAP measurements available at both PDNA initiation and the specified post-administration time point were included. Patients missing either measurement were excluded from the comparison. Consequently, the number of patients included could differ between comparisons. These analyses were considered hypothesis-generating; therefore, no adjustment was made for multiple comparisons. All analyses were performed using R version 4.5.0 (The R Foundation for Statistical Computing, Vienna, Austria).

## Results

### Patient characteristics

**Fig. 1:**
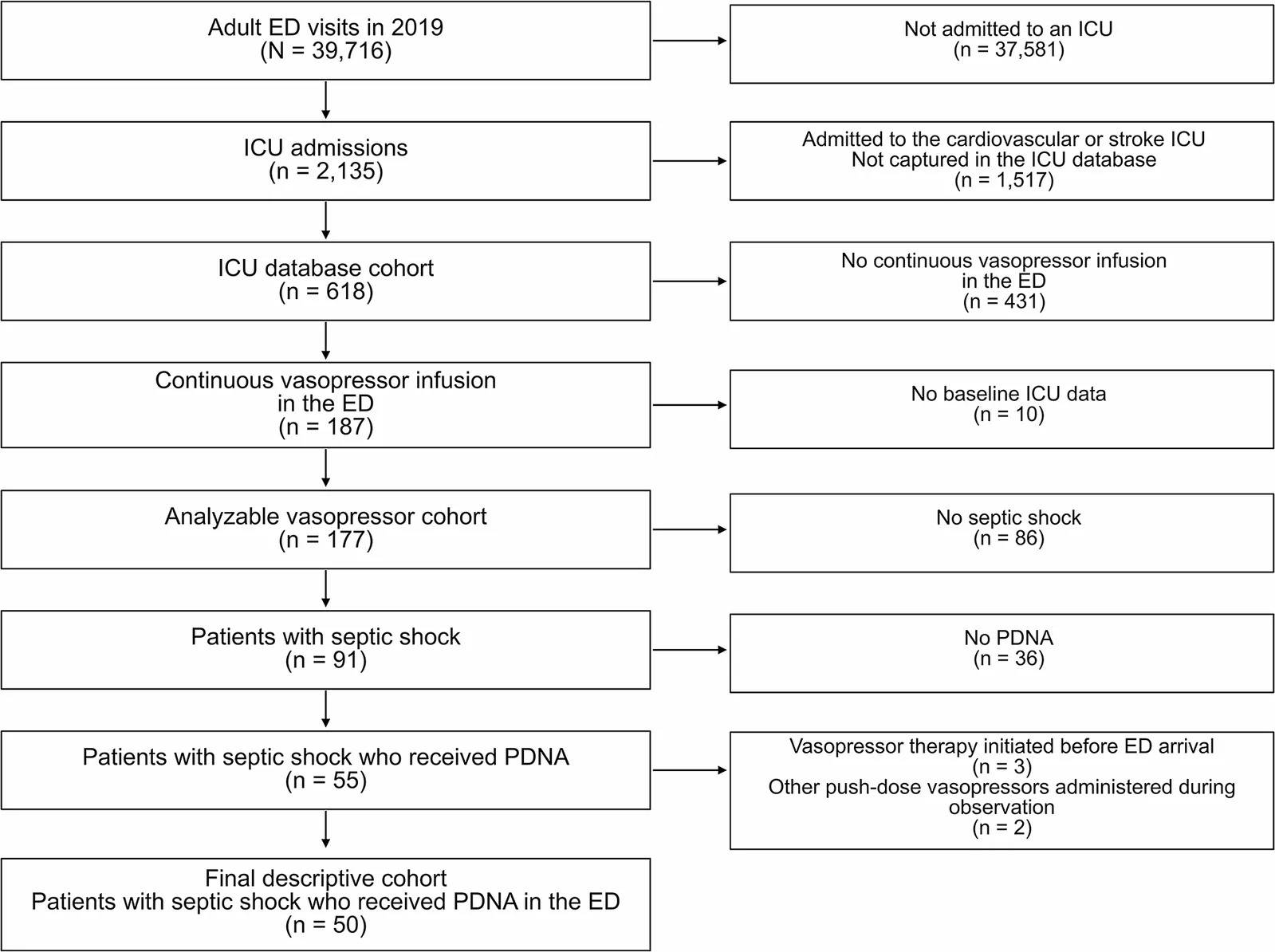
Flow diagram of patient selection. Among 39,716 adults with emergency department visits in 2019, patients were excluded according to the prespecified eligibility criteria. The final descriptive cohort comprised 50 patients with septic shock who received push-dose noradrenaline in the emergency department and were subsequently admitted to an ICU. Abbreviations: ED, emergency department; ICU, intensive care unit; PDNA, push-dose noradrenaline

During 2019, 39,716 adults (≥ 18 years) visited the ED. Patients who were not admitted to an ICU (*n* = 37,581), patients admitted to cardiovascular or stroke-specific ICUs not captured in the ICU database (*n* = 1,517), patients who did not receive continuous vasopressor administration in the ED (*n* = 431), patients with missing essential baseline data (*n* = 10), and patients without septic shock (*n* = 86) (Fig. 1) were excluded. Of the remaining 91 patients with septic shock, 50 received PDNA in the ED, were admitted to an ICU, and were included in the analysis. The patient characteristics are shown in Table 1.

Among the 50 patients who received PDNA, the median (IQR) age was 78 (67–84) years. Among the 30 patients with MAP measurements at PDNA initiation, the directly observed median (IQR) MAP at PDNA initiation was 52 (44–61) mmHg. At ICU admission, the median (IQR) APACHE II score was 23 (19–32), and the median (IQR) blood lactate level was 3.4 (2.1–7.5) mmol/L.

**Table 1:** Patient characteristics and clinical status at push-dose noradrenaline initiation

Variables	PDNA (*N* = 50)	
*Baseline characteristics*		
Age (years), median (IQR)	78 (67–84)	
Sex, n (%)		
Male	23 (46)	
Female	27 (54)	
Diagnostic category		
Respiratory disease, n (%)	10 (20)	
Gastrointestinal disease, n (%)	20 (40)	
Genitourinary disease, n (%)	16 (32)	
Metabolic disease, n (%)	1 (2)	
Other, n (%)	3 (6)	
CCI, median (IQR)	6 (4–7)	
Physiological parameters on ED arrival		
SBP (mmHg), median (IQR)	95 (77–126)	
DBP (mmHg), median (IQR)	52 (41–65)	
MAP (mmHg), median (IQR)	66 (57–81)	
Heart rate (beats/min), median (IQR)	112 (96–125)	
GCS, median (IQR)	14 (9–15)	
*At initial PDNA administration*		
Arterial-line monitoring at PDNA initiation, n (%)	23 (46)	
Directly observed MAP at PDNA initiation, median (IQR) (*n* = 30)	52 (44–61)	
Total fluid volume administered from ED arrival to PDNA initiation (mL), median (IQR)	750 (500–1462)	

Unless otherwise specified, percentages were calculated using the full cohort as the denominator (*n* = 50). Continuous variables were summarized among patients with available data; the number of missing observations is shown where applicable

CCI, Charlson Comorbidity Index; DBP, diastolic blood pressure; ED, emergency department; GCS, Glasgow Coma Scale; IQR, interquartile range; MAP, mean arterial pressure; PDNA, push-dose noradrenaline; SBP, systolic blood pressure

### PDNA use

PDNA use and early vasopressor management are summarized in Table 2. During the 15-minute observation window, PDNA was the only push-dose vasopressor administered, and all 50 patients received CNI as the sole continuous vasopressor therapy. The median (IQR) initial CNI rate was 300 (150–300) µg/h. Among the 50 patients, 25 (50%) received PDNA at CNI initiation, 6 (12%) during CNI dose escalation, and 19 (38%) as rescue administration before CNI initiation. These predefined administration contexts corresponded to PDNA administration while CNI was being established, during escalation of ongoing infusion, and before continuous infusion had been initiated, respectively.

**Table 2:** Push-dose noradrenaline use, early vasopressor management, and clinical outcomes

Variables	PDNA (*N* = 50)
PDNA administration context	
At CNI initiation, n (%)	25 (50)
During CNI dose escalation, n (%)	6 (12)
Rescue administration before CNI initiation, n (%)	19 (38)
PDNA administration and early vasopressor management	
Number of boluses of PDNA received during the 15-minute observation window, n (%)	
1	32 (64)
2	14 (28)
3	3 (6)
4	1 (2)
*0–5-minute window*	
First bolus dose (µg), median (IQR)	20 (20–30)
Initial CNI rate (µg/h), median (IQR)	300 (150–300)
*5–15-minute window*	
PDNA dose per bolus (µg), median (IQR), *n* = 12	20 (20–35)
Active CNI rate (µg/h), median (IQR)	300 (150–575)
*On ICU admission*	
APACHE II score, median (IQR)	23 (19–32)
Blood lactate level (mmol/L), median (IQR)	3.4 (2.1–7.5)
*During hospital stay*	
In-hospital mortality, n (%)	10 (20)

Unless otherwise specified, percentages were calculated using the full cohort as the denominator (*n* = 50). Continuous variables were summarized among patients with available data; the number of missing observations is shown where applicable. The CNI rate was treated as 0 before CNI initiation. All noradrenaline doses are reported as noradrenaline base according to the labeling of the formulation used

Abbreviations: APACHE, Acute Physiology and Chronic Health Evaluation; CNI, continuous noradrenaline infusion; ICU, intensive care unit; IQR, interquartile range; PDNA, push-dose noradrenaline

In addition, PDNA administration was temporally associated with endotracheal intubation in 9 patients (18%). The median (IQR) first PDNA dose was 20 (20–30) µg. During the 15-minute observation window, 32 (64%) patients received one bolus, 14 (28%) received two, 3 (6%) received three, and 1 (2%) received four.

**Fig. 2:**
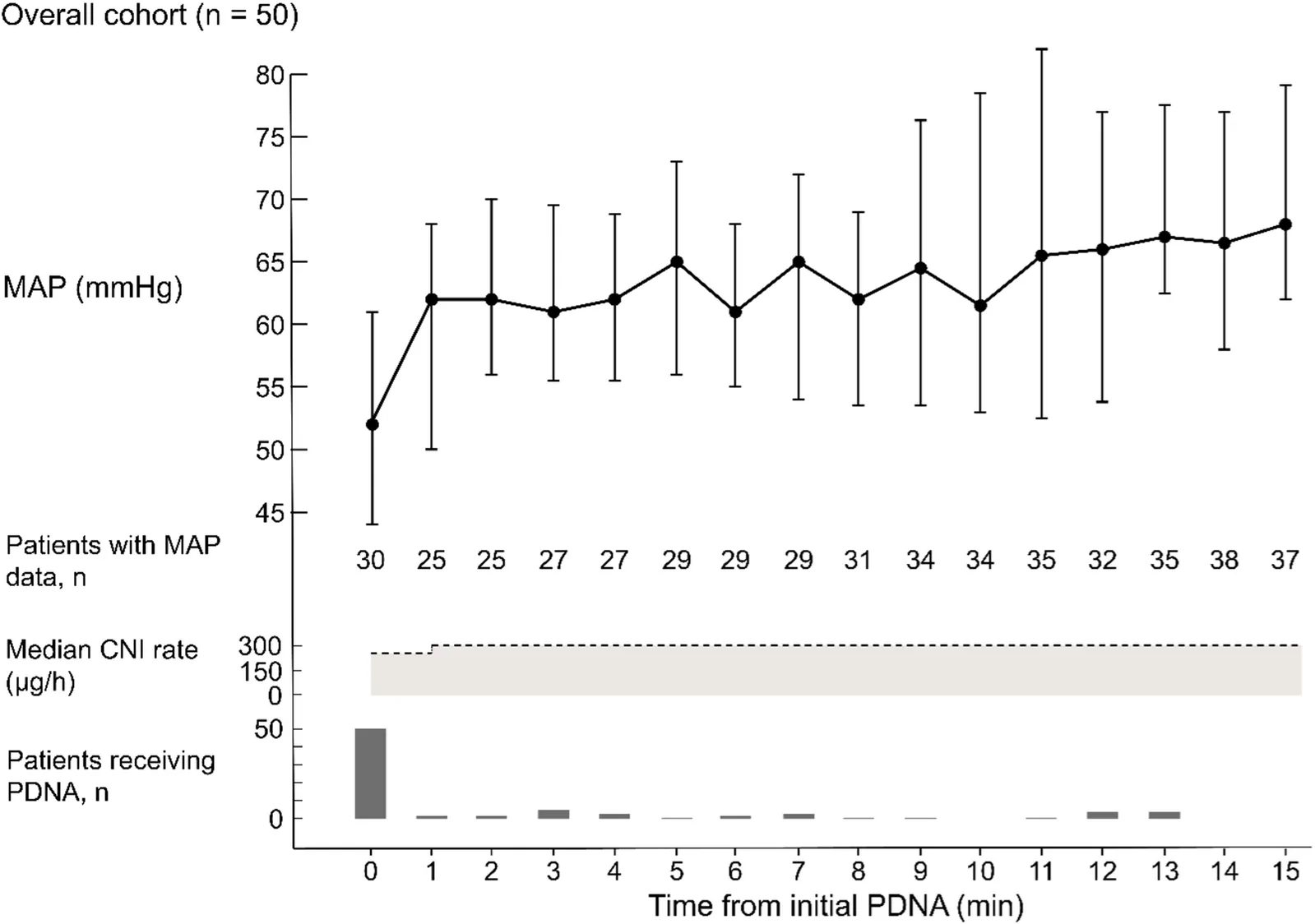
Vasopressor administration and changes in mean arterial pressure (MAP) during the 15-minute observation window. The observation period started at the time of the initial PDNA administration. The black line with filled circles shows the median MAP, and vertical error bars indicate the interquartile range. Numbers below the MAP curve indicate the number of patients contributing directly observed MAP data at each time point. MAP values were based on directly observed measurements without interpolation or nearest-value substitution; therefore, the number of patients contributing MAP data varied across time points. The light-gray stepped area with its upper boundary indicated by a dashed line shows the median CNI rate. The dark-gray bars at the bottom indicate the number of patients receiving PDNA at each minute and correspond to the lower inline PDNA scale on the left. Abbreviations: CNI, continuous noradrenaline infusion; MAP, mean arterial pressure; PDNA, push-dose noradrenaline

**Fig. 3:**
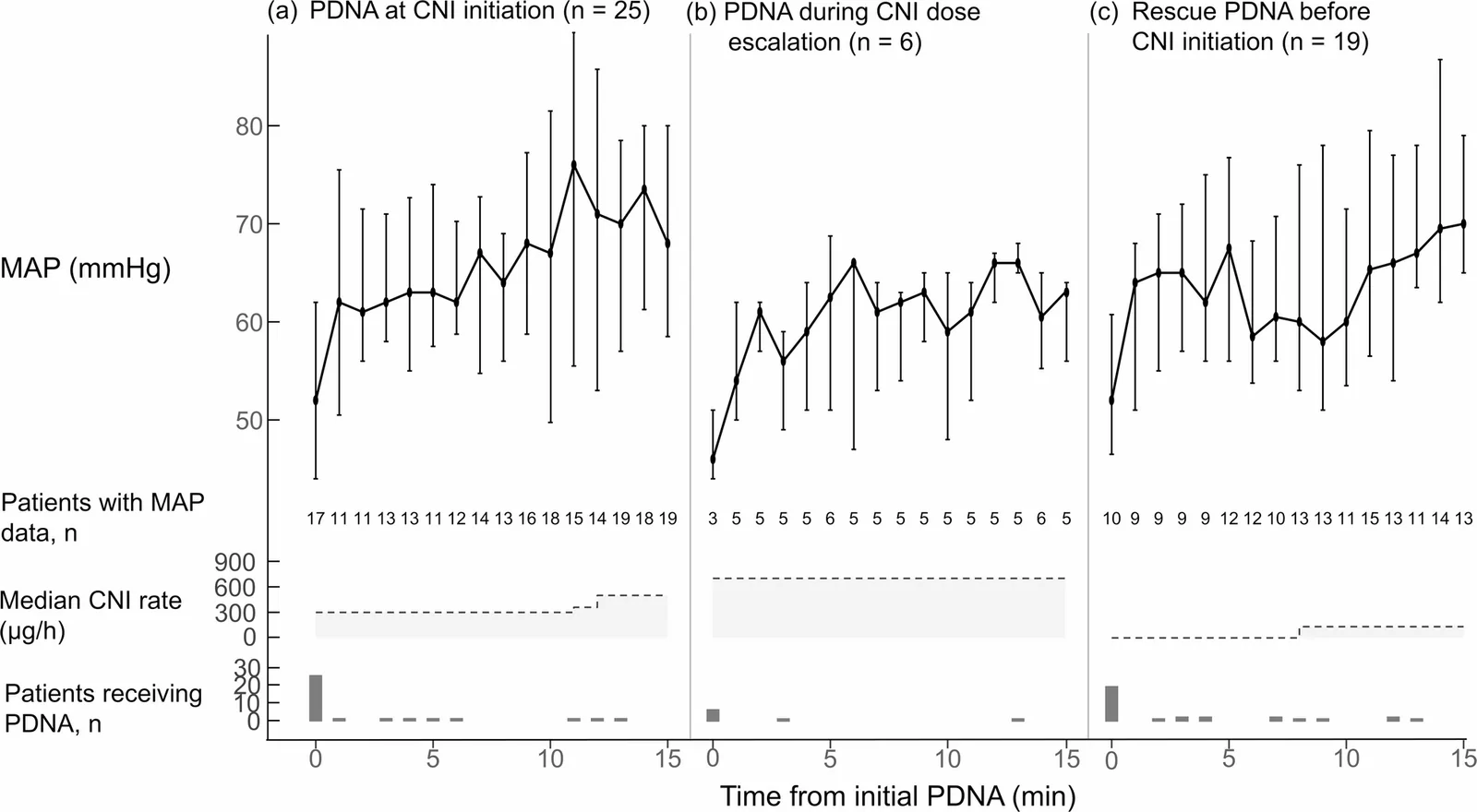
Observed MAP changes and concurrent vasopressor administration according to PDNA administration context. (**a**) PDNA at CNI initiation, (**b**) PDNA during CNI dose escalation, and (**c**) rescue PDNA before CNI initiation. The observation period started at the time of the initial PDNA administration. The black line with filled circles shows the median MAP, and the vertical error bars indicate the interquartile range. Numbers below the MAP curve indicate the number of patients contributing directly observed MAP data at each time point. MAP values were based on directly observed measurements without interpolation or nearest-value substitution; therefore, the number of patients contributing MAP data varied by time point. The light-gray stepped areas, with their upper boundaries indicated by dashed step lines, show the median CNI rates and correspond to the shared CNI scale on the left. The dark-gray bars at the bottom indicate the number of patients receiving PDNA at each minute and correspond to the shared PDNA scale on the left. Because the CNI dose-escalation subgroup included only six patients, the findings shown in panel (**b**) should be interpreted with caution. Abbreviations: CNI, continuous noradrenaline infusion; MAP, mean arterial pressure; PDNA, push-dose noradrenaline

Directly observed MAP values were available for 30 patients at PDNA initiation and for a variable number of patients at each subsequent time point (Fig. 2). The directly observed median (IQR) MAP values at 1, 5, 10, and 15 min were 62 (50–68), 65 (56–73), 62 (53–79), and 68 (62–79) mmHg, respectively. The number of patients contributing data at each time point is reported in Supplementary Table S1. In exploratory paired analyses restricted to patients with directly observed MAP measurements available both at PDNA initiation and at each respective post-administration time point, MAP values at 1, 5, 10, and 15 min were higher than those at PDNA initiation (paired *n* = 17, 21, 23, and 25, respectively; *p* = 0.017, 0.002, 0.004, and < 0.001, respectively). Descriptive MAP trajectories according to the context of PDNA administration are shown Fig. 3. Of the 50 patients, 14 had insufficient blood pressure and/or HR data within the prespecified assessment window to evaluate physiological adverse events, leaving 36 patients in whom physiological adverse events could be evaluated.Among these 36 patients, 1 (3%) experienced an increase in HR of > 30%. The event required no treatment and resolved within 3 min. Among all 50 patients, no medication errors or serious adverse events were documented.

## Discussion

This study characterized the clinical contexts of PDNA use, early MAP trajectories, and prespecified adverse events after PDNA administration in patients presenting to the ED with septic shock. Although PDNA was most commonly administered at the time of CNI initiation, it was also administered during CNI dose escalation or as rescue administration before CNI initiation in some cases. During the 15-minute observation period, an early increase in median MAP was observed across all three administration contexts. These findings describe the use of PDNA during the early phase of CNI management. However, because CNI initiation or titration, fluid administration, and other resuscitative interventions occurred concurrently, the observed MAP changes cannot be attributed solely to PDNA administration.

The PDNA doses used in our cohort were higher than those reported in perioperative studies, which typically range from 4 to 16 µg [7]. In contrast, in our cohort, the median initial PDNA dose was 20 µg, and some patients received additional boluses. However, this cross-study comparison should be interpreted with caution because the patient populations and clinical settings differed. Because this study did not assess the reasons underlying dose selection or compare different PDNA doses, the observed dosing pattern should not be interpreted as evidence that higher doses are required in septic shock or as indicating an optimal dose. These findings highlight the need for prospective dose-ranging studies to determine whether optimal PDNA dosing differs according to the clinical setting.

One possible explanation for the observed early increase in MAP is that PDNA may have produced a transient loading-dose-like effect during the initial phase of CNI. From a pharmacokinetic–pharmacodynamic perspective, a bolus could transiently increase noradrenaline exposure and allow an effective concentration to be reached more rapidly than with infusion alone [14]. However, this interpretation is speculative because plasma noradrenaline concentrations were not measured and this study did not include a CNI-alone comparison group. Nishikimi et al. [5] reported a dose-dependent early MAP response after CNI initiation, with MAP slopes during the first 15 min of 0.37 mmHg/min with 0.05 µg/kg/min and 0.51 mmHg/min with 0.10 µg/kg/min, suggesting that the pressor response to CNI alone may be gradual or insufficient during the early phase, particularly when the starting dose is low and baseline hypotension is severe. However, these findings do not establish whether adding PDNA to CNI provides additional benefit. Prospective pharmacokinetic and comparative studies are warranted to determine whether PDNA provides additional early hemodynamic support beyond that provided by CNI and other concurrent interventions.

Among the 36 patients in whom physiological adverse events could be evaluated, one experienced an increase in HR of > 30% without any prespecified extreme blood pressure response. Among all 50 patients, no serious adverse events or medication errors were documented. However, adverse events were identified retrospectively from routinely collected clinical data; therefore, transient hypertension, arrhythmias, ischemic events, or unrecognized medication preparation errors may have been missed. No patients experienced prespecified extreme blood pressure responses, including an SBP > 200 mmHg or an SBP increase of > 100% from baseline. The low frequency of a > 30% increase or decrease in HR is consistent with the expected pharmacologic profile of noradrenaline, but the limited and retrospective safety assessment precludes firm conclusions [4, 7, 12, 13, 15].

To our knowledge, this is the first study to describe the contexts and patterns of PDNA use and early hemodynamic trajectories in patients with septic shock in the ED [6, 7]. This retrospective, noncomparative study provides descriptive data that may inform hypotheses regarding PDNA use during early CNI management, but it does not establish the effectiveness or safety of PDNA. Larger prospective comparative studies with standardized monitoring and treatment protocols are warranted to establish the safety and effectiveness of PDNA administration in patients with septic shock in the ED setting.

This study has several limitations. First, it was a single-center, retrospective, descriptive study with a small sample size. Because the data were collected in 2019, subsequent changes in vasopressor practice, monitoring, and sepsis care may limit applicability to contemporary ED practice; therefore, these results should be validated in a more recent cohort. Second, PDNA was administered at the treating clinician’s discretion, generally combined with fluid administration and CNI initiation or titration. Therefore, the observed MAP changes cannot be attributed solely to PDNA administration. However, this heterogeneity reflects the way PDNA is used in routine emergency care. Third, blood pressure measurements were not standardized, and both invasive and noninvasive measurements were used. This may have introduced measurement variability, although it also reflects real-world ED practice. Fourth, because early MAP trajectories were derived retrospectively from routinely obtained measurements, the findings may be biased because of missing observations. However, the direction and magnitude of any resulting bias are unknown because the pattern of missing measurements could not be determined. The number of patients contributing MAP measurements at each time point is reported in the Results section and Supplementary Table S1 to improve transparency. Fifth, missing blood pressure or HR data reduced the number of patients who could be evaluated for adverse events, and this may have led to incomplete ascertainment of safety signals. Although no serious adverse events or medication errors were documented, these safety findings should be considered preliminary. Finally, the short observation window of 15 min and lack of a control group limit our ability to assess sustained hemodynamic effects, downstream outcomes, and the comparative effectiveness of PDNA compared with CNI alone.

## Conclusions

In this single-center, retrospective, descriptive study of patients with septic shock in the ED, PDNA was most commonly used at the time of CNI initiation. PDNA use was followed by an early increase in median MAP in all administration contexts considered. No serious adverse events or medication errors were documented; however, safety assessment was limited owing to missing data and retrospective ascertainment. These findings do not establish the effectiveness or safety of PDNA; therefore, larger prospective comparative studies with standardized monitoring are warranted. 

## Supplementary Information

Below is the link to the electronic supplementary material.

Supplementary Material 1

## Data Availability

The datasets used and/or analyzed in this study are available from the corresponding author upon reasonable request.

## Abbreviations

APACHE: Acute physiology and chronic health evaluation
BP: Blood pressure
CCI: Charlson comorbidity index
CNI: Continuous noradrenaline infusion
DBP: Diastolic blood pressure
ED: Emergency department
EMR: Electronic medical record
GCS: Glasgow coma scale
HR: Heart rate
ICU: Intensive care unit
IQR: Interquartile range
MAP: Mean arterial pressure
PDNA: Push-dose noradrenaline
SBP: Systolic blood pressure
