# Supplementary material for: Early hemodynamic changes after the use of push-dose noradrenaline in patients presenting to the emergency department with septic shock: a descriptive study
Source: BMC Emerg Med. 2026 Sep 19;26:274. doi: 10.1186/s12873-026-01773-8 (PMC13685283; doi:10.1186/s12873-026-01773-8)
Supplement: Supplementary file 1 [file 12873_2026_1773_MOESM1_ESM.docx]

**Supplementary Table S1. Availability of directly observed MAP and HR measurements around initial PDNA administration**

**Panel A. Directly observed MAP measurements from PDNA initiation through 15 minutes**

| **Time from initial PDNA, min** | **MAP, median (IQR), mmHg** | **Patients with directly observed MAP data, n** | **Patients with paired MAP data, n** |
| --- | --- | --- | --- |
| 0 | 52 (44–61) | 30 | NA |
| 1 | 62 (50–68) | 25 | 17 |
| 2 | 62 (56–70) | 25 | 20 |
| 3 | 61 (56–70) | 27 | 23 |
| 4 | 62 (56–69) | 27 | 18 |
| 5 | 65 (56–73) | 29 | 21 |
| 6 | 61 (55–68) | 29 | 22 |
| 7 | 65 (54–72) | 29 | 20 |
| 8 | 62 (54–69) | 31 | 22 |
| 9 | 65 (54–76) | 34 | 22 |
| 10 | 62 (53–79) | 34 | 23 |
| 11 | 65 (53–82) | 35 | 22 |
| 12 | 66 (54–77) | 32 | 20 |
| 13 | 67 (63–78) | 35 | 22 |
| 14 | 67 (58–77) | 38 | 23 |
| 15 | 68 (62–79) | 37 | 25 |

**Panel B. HR measurements before and after initial PDNA administration**

| **HR measurement** | **HR, median (IQR), beats/min** | **Patients with available HR data, *n*** | **Patients with paired HR data, *n*** |
| --- | --- | --- | --- |
| Before initial PDNA | 104 (94–120) | 44 | NA |
| After initial PDNA | 106 (89–115) | 43 | 42 |

MAP values were based on blood pressure measurements recorded at the specified minute during routine clinical care, without imputation, interpolation, or nearest-time substitution. The patients contributing MAP data varied across time points. Paired MAP sample sizes represent patients with MAP values available both at PDNA initiation and at the specified post-administration time point. Baseline HR was defined as the last recorded value before the initial PDNA administration. The post-PDNA HR value represents the measurement showing the greatest absolute change from baseline within 5 minutes after administration. The paired HR sample size represents patients with HR measurements available both before and after the initial PDNA administration. The timing of the post-PDNA HR measurement was not standardized.

Abbreviations: HR, heart rate; IQR, interquartile range; MAP, mean arterial pressure; NA, not applicable; PDNA, push-dose noradrenaline.
